# Patterns and risk factors of pig transport mortality: implications for official controls in a high-throughput slaughterhouse

**DOI:** 10.1186/s40813-026-00519-z

**Published:** 2026-05-07

**Authors:** Alfonso Rosamilia, Claudia Weiss, Francesca Iacoponi, Chiara Casadio, Chiara Guarnieri, Stefano Benedetti, Giuseppe Marruchella, Giuseppe Merialdi

**Affiliations:** 1 Istituto Zooprofilattico Sperimentale della Lombardia e dell’Emilia- Romagna, Via Antonio Bianchi 9, 25124 Brescia, Italy; 2 Regione Emilia-Romagna, Settore Prevenzione Collettiva and Sanità Pubblica, Viale Aldo Moro 21, 40127 Bologna, Italy; 3 Department of Food Safety, Nutrition and Veterinary Public Health, Istituto Superiore di Sanità, Viale Regina Elena 299, 00161 Rome, Italy; 4 Local Health Unit Authority, Via San Giovanni del Cantone 23, 41121 Modena, Italy; 5 Department of Veterinary Medicine, University of Teramo, Località Piano d’Accio, 64100 Teramo, Italy

**Keywords:** Pig welfare, Transport mortality, Journeys lasting ≤ 8 hours, Ambient temperature, Season, Official control

## Abstract

**Background:**

Transport to slaughterhouses is a critical phase in pig production, affecting welfare and economics. Mortality during transport, or dead-on-arrival (DOA), is a direct indicator of welfare compromise. This study evaluated the association between estimated transport distance, ambient temperature, and consignment size with mortality in commercial pig journeys lasting ≤ 8 h.

**Results:**

Data from 34,239 consignments from 434 farms transported to a high-throughput slaughterhouse were analyzed. Consignment size ranged from 5 to 164 pigs, estimated transport distances from 5 to 605 km, and ambient loading temperatures ranged from 0.9 °C to 31.2 °C. Overall average mortality rate was 0.062%, with 93.1% of consignments showing no DOA pigs. Seasonal patterns were pronounced, with summer exhibiting the highest mortality and winter/spring the lowest. Mixed-effects logistic regression showed that ambient temperature and estimated transport distance were positively associated with mortality (OR = 1.354 and 1.086, respectively), while larger consignments exhibited a negative association (OR = 0.910). Each 1 °C increase in temperature corresponded to a 3.86% rise in the odds of pigs being DOA. Random farm effects showed substantial between-farm variability. Average transport mortality per farm categorized 36.9% of farms as being at very low risk, 13.1% as low, 24.9% as medium, and 25.1% as high risk. Best Linear Unbiased Predictors (BLUP) estimates identified 38 farms with significantly higher-than-average mortality, while funnel plot analysis highlighted 26 outliers, providing complementary approaches to benchmark farm performance and prioritize high-risk holdings for welfare monitoring. Among 664 (1.95%) consignments inspected by official veterinarians, mortality was 0.036%, and inspections were evenly distributed across seasons, ambient temperatures, and consignment characteristics. Only 86 (12.95%) of inspections targeted the 26 high-risk farms identified by the funnel plot. These results suggest that inspection efforts were not preferentially directed toward farms with elevated predicted mortality.

**Conclusions:**

Pig mortality during journeys lasting ≤ 8 h transports is mainly associated with ambient temperature and, to a lesser extent, estimated transport distance, with summer as a high-risk period. Larger consignments modestly reduce risk, and mortality per farm highlight the potential for targeted interventions. Current veterinary inspections are not systematically aligned with risk, suggesting scope to optimize monitoring and welfare outcomes.

**Supplementary Information:**

The online version contains supplementary material available at 10.1186/s40813-026-00519-z.

## Background

Transport of pigs to slaughterhouses is a critical phase in the swine production chain, with major implications for both animal welfare and economic sustainability [1, 2]. Pigs are particularly susceptible to transport-related stressors, which may result in injuries, fatigue, impaired meat quality, and mortality [3–5]. While transport stress encompasses a wide spectrum of physiological and behavioral disturbances, mortality during transport (defined as dead-on-arrival, DOA) represents the most severe and readily measurable indicator of welfare failure during this phase. In a survey conducted across five European countries, the average mortality rate during pig transport was reported to be 0.11% [6]. Although this proportion appear low, even small losses may have considerable economic impact; for example, pre-slaughter losses in Italian heavy pigs were estimated at €463,000 over a two-year period in a recent study [7].

In the European Union (EU), the protection of animals during transport is regulated by Council Regulation (EC) No 1/2005, which establishes specific requirements according to journey duration. Journeys are classified as either ≤ 8 h or > 8 h, with additional provisions applying to longer journeys [8]. In Italy, compliance with these requirements in monitored through the “National Animal Welfare Plan” (“Piano Nazionale Benessere Animale,” PNBA), which adopts a risk-based inspection strategy and requires veterinary controls on at least 10% of long journeys and 2% of journeys lasting ≤ 8 h, particularly during periods of climatic risk such as summer and winter [9]. Despite these framework, variability in enforcement and data collection across Member States continues to limit harmonized implementation and evaluation of transport welfare across the EU. Differences in inspection procedures, interpretation of fitness-for-transport criteria, and the lack of harmonised digital data recording systems generate fragmented datasets, which restrict cross-country comparisons and hinder the development of standardised risk-assessment models [10].

Transport stress results from the interaction of multiple factors, including the physical condition of animals at loading, environmental and vehicle microclimatic conditions (temperature, humidity, ventilation), handling practices, stocking density, vibrations, abrupt vehicle movements, and journey duration [11–13]. Genetic selection also contributes to transport vulnerability. Modern high-performance pig genotypes, selected for rapid growth and lean meat production, generate greater metabolic heat compared with earlier genetic lines, which reduces their ability to cope with thermal challenges during transport [14]. In addition, vehicle design and road conditions are also related to welfare outcomes; adequate ventilation systems, suspension, and compartmentalization can mitigate stress, whereas poor vehicle design increases the risk of injuries and thermal stress [12, 15, 16]. Handling practices and mixing pigs from different farms may further exacerbate aggression and carcass downgrading [17].

Experimental studies have shown that journey duration is a major determinant of transport-related welfare deterioration. In cull sows, for example, clinical conditions such as gait scores and physical energy levels progressively worsen as journey duration increases from 4 to 8 h [18]. Behavioral observations during transport also indicate that animals exhibit increasing signs of fatigue and reduced capacity to cope with the vehicle’s microclimate as the journey progresses, challenging the hypothesis that pigs recover physiologically during transit [19]. Moreover, pigs typically remain standing during the initial 20–30 min of transport to maintain balance, a phase characterized by increased physical exertion at the start of the journey [20].

Seasonal and environmental conditions play a central role in transport outcomes. High ambient temperatures during summer are consistently associated with increased mortality, heat stress, and reduced meat quality, particularly in heavy pigs [21–23]. Conversely, cold winter conditions may lead to hypothermia, carcass lesions, and muscle defects such as red, soft and exudative (RSE) meat [23]. Susceptibility to these conditions also varies according to animal characteristics and journey duration. Lighter pigs appear more vulnerable during long-distance transport (8–24 h) [12], whereas heavier pigs may be particularly susceptible even during relatively short journeys, for example around 90 min [24]. This vulnerability may be related to acute stress and overheating during loading and the early phases of transport, which may stabilize once the vehicle microclimate reaches a steady state. In Italy, pig production is largely oriented towards heavy animals (160–170 kg live weight) destined for Protected Designation of Origin (PDO) products, which may further increase susceptibility to thermal stress due to limited thermoregulatory capacity and higher metabolic heat production [25].

Recognising the complexity of welfare challenges associated with animal transport, the European Commission recently initiated a revision of animal welfare legislation under the Farm to Fork Strategy, supported by scientific assessments from the European Food Safety Authority (EFSA) [14]. In its 2022 Scientific Opinion, the EFSA Panel on Animal Health and Welfare (AHAW) identified several critical welfare consequences associated with the transit stage in pigs, including heat stress, motion stress, and injuries. These outcomes are primarily linked to hazards such as inadequate microclimatic control, unsuitable vehicle design, and inappropriate handling practices. The Opinion also proposed quantitative thresholds for finishing pigs, indicating a thermal comfort zone up to 22 °C and an upper critical temperature of 25 °C.

Despite the recognized importance of factors such as transport distance, ambient temperature, and loading density, quantitative analyses examining their combined effects under commercial transport conditions remain limited [10]. In particular, evidence on the interaction between journey distance and thermal conditions is still scarce, restricting the development of effective risk-based mitigation strategies.

This observational study aimed to investigate the associations between estimated transport distance, ambient temperature, and mortality during commercial pig journeys lasting ≤ 8 h. Using five years of routinely collected data (2020–2024) from a high-throughput Italian slaughterhouse, the study seeks to provide evidence to support risk-based inspection strategies and targeted welfare interventions during periods of increased thermal risk.

## Materials and methods

### Data collection

The study was conducted at a high-throughput slaughterhouse located in the Emilia-Romagna region of northern Italy (44°45′ N; 11°00′ E), processes approximately 850,000 pigs annually. The majority of animals were heavy pigs (≥ 9 months of age; average live weight ≈ 170 kg), born and raised in Italy within the Prosciutto di Parma production chain.

**Fig. 1:**
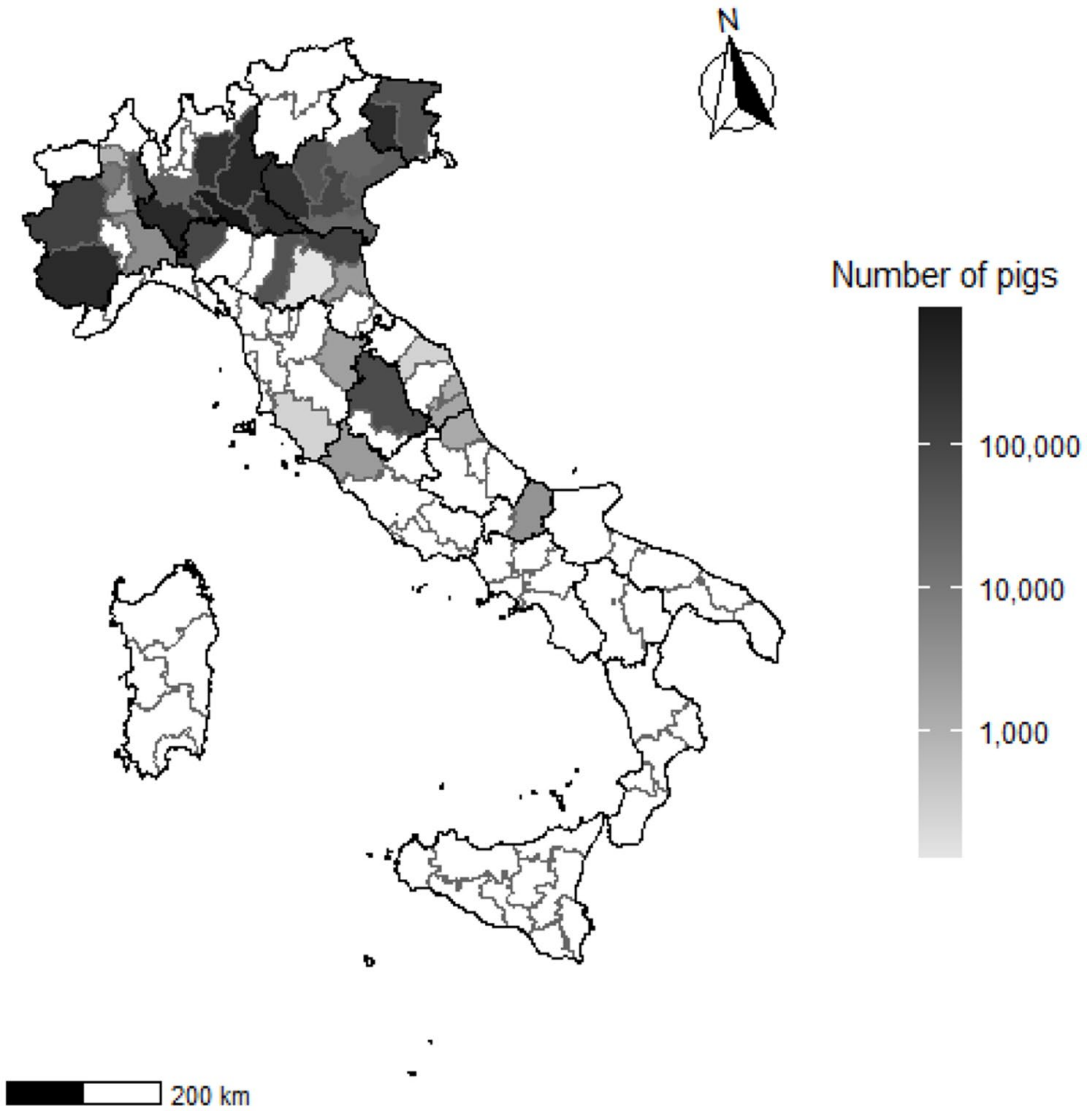
Map showing the number of pigs (*n* = 4,293,698) slaughtered in the period 2020–2024 by the surveyed slaughterhouse, divided per province in Italy

Between January 2020 and December 2024, a total of 34,239 consignments were analyzed, corresponding to 4,293,698 pigs originating from 434 farms across 36 Italian provinces (Fig. 1). Most consignments originated from northern Italy, particularly Lombardy (58.15%), followed by Piedmont (12.96%), Veneto (10.67%), Friuli–Venezia Giulia (8.54%), and Emilia–Romagna (7.72%). Contributions from central and southern regions were limited (Umbria 1.71%; Molise 0.07%; Marche 0.06%; Lazio 0.05%; Tuscany 0.05%; Abruzzo 0.03%).

All pigs were transported on journeys lasting ≤ 8 h using vehicles authorized for live animal transport in accordance with Council Regulation (EC) No 1/2005 [8]. Data were retrospectively obtained from slaughterhouse records and official transport inspection reports. Each record corresponded to a single consignment (farm-to-slaughter delivery) on a specific date. The following data were systematically recorded: slaughter date, farm identification code, consignment size (number of pigs), and number of DOA pigs.

Farm identification codes were anonymized prior to analysis to ensure confidentiality. Seasons were defined as spring (March–May), summer (June–August), autumn (September–November), and winter (December–February). Ambient temperature at loading was defined as the average monthly temperature at the farm location, obtained from official meteorological data [26]. As exact journey durations were not available, estimated transport distance (km) was used as a proxy for journey duration. Distances were calculated using Google Maps by selecting the shortest available road route between the farm and the slaughterhouse [27].

### Statistical analysis

A set of explanatory variables was considered, including continuous variables (estimated transport distance, ambient temperature at loading, consignment size, and mortality proportion) and categorical variables (season and farm) (Table S1). Continuous variables were summarized using mean and standard deviation (SD), median, range (minimum–maximum), and interquartile range (IQR). The normality of distributions was assessed using the Anderson–Darling test.

Mortality was expressed both as the number of DOA pigs per consignment and as the proportion of pigs transported. Associations between mortality proportion and continuous predictors were explored using Spearman’s rank correlation coefficient. Differences across seasons were assessed using the Kruskal–Wallis test, followed by Dunn’s post hoc test with Bonferroni adjustment for multiple comparisons.

Univariable logistic regression models were fitted to evaluate the association between mortality at consignment level (binary outcome: absence vs. presence of ≥ 1 DOA pig) and each explanatory variable. For the categorical variable “season”, autumn was used as the reference category. Results are presented as odds ratios (ORs) with 95% confidence intervals (CIs) and corresponding P-values. Statistical significance was set at *P* < 0.05.

Prior to inclusion of random effects, a multivariable Poisson regression model was fitted to explore the combined effects of the explanatory variables. Multicollinearity was assessed by calculating variance inflation factors (VIFs), and pairwise correlation matrices were examined to verify the independence of predictors.

### Mixed-effects modelling

Generalised linear mixed models (GLMMs) were used to account for the hierarchical structure of the data, with multiple consignments originating from the same farms over time. This approach allows the inclusion of both fixed and random effects, thereby avoiding pseudoreplication and appropriately modelling within-farm correlation [28]. Farm of origin was included as a random intercept to account for clustering per farm.

Given the binomial nature of the outcome, the model was fitted with a logit link function to assess the association between transport-related factors and mortality risk. The response variable was specified as the number of DOA pigs relative to the total number of pigs transported per consignment. Continuous predictors were standardised (z-scores) prior to analysis.

The model was specified as:

$$\begin{aligned}\mathrm{logit}(p_\mathrm{ij}) = &\:\beta_{0} +\beta_{1} \mathrm{km}_\mathrm{z}+\beta_{2}\:\mathrm{temp}\_\mathrm{load}_z\cr & \quad\beta_{3}\:\mathrm{n}\_\mathrm{pigs}_z + u_{j}\end{aligned}$$

where *u*_*j*_ ∼ *N*(0,σ^2^_farm_) represents the random effect of farm 𝑗. The model was fitted using glmer() (lme4) [29] with the “bobyqa” optimizer and up to 200,000 iterations. Model performance and goodness-of-fit were assessed via marginal and conditional R² (performance), convergence diagnostics, and residual simulation using the DHARMa package [30]. Residual diagnostics included assessment of residual uniformity using the Kolmogorov–Smirnov test, evaluation of overdispersion, and identification of potential outliers. Coefficients were exponentiated to obtain ORs and 95% Wald CIs.

To account for the high proportion of zero-mortality consignments (~ 93%), a zero-inflated binomial (ZIB) model was additionally fitted using the glmmTMB package [31]. The ZIB model included the same fixed and random effects as the GLMM, with an additional zero-inflation component. Continuous predictors were standardized as z-scores, and ORs for temperature were back-transformed to represent a 1 °C increase. ORs from the conditional component of the model represent the effect of each covariate on mortality probability, conditional on non-zero mortality. Model selection was based on the Akaike Information Criterion (AIC), with the model showing the lowest AIC retained.

To assess the robustness of the results, a bootstrap-based power analysis was performed for each fixed effect. For both GLMM and ZIB models, 200 bootstrap datasets were generated by resampling consignments within farms. Models were refitted on each bootstrap sample, and statistical significance of each predictor was assessed at α = 0.05. Statistical power was estimated as the proportion of bootstrap samples in which each effect was significant, with 95% confidence intervals derived from the empirical distribution. This approach accounts for both the hierarchical structure of the data and the excess of zero outcomes.

### Farm-level risk analysis

Between-farm variability in transport mortality was investigated to identify holdings potentially associated with increased risk. The average transport mortality per farm was defined as the ratio between the total number of pigs DOA and the total number of pigs transported from each farm during the study period. This value was used to classify holdings into four exploratory risk categories: very low (≤ 25th percentile), low (> 25th to 50th), medium (> 50th to 75th), and high risk (> 75th percentile of the mortality distribution).

To complement the mixed-effects modelling, empirical Bayes estimates (Best Linear Unbiased Predictors, BLUPs) of the farm-specific random intercepts were extracted from the GLMM to quantify deviations from the overall mean mortality risk. Farms with BLUP estimates and corresponding 95% CIs entirely above zero were interpreted as having a higher-than-average mortality risk. Differences across risk categories were visualized using box plots of average mortality per farm.

Additionally, funnel plots of farm-level mortality rates with 95% and 99.8% binomial control limits were generated to visually identify potential statistical outliers, considering the number of animals transported per farm. Statistical significance was defined as *P* < 0.05 for fixed effects, and farm-specific deviations were interpreted via BLUP CIs.

In addition, funnel plots were constructed to assess farm-level mortality rates in relation to the number of pigs transported per farm. Control limits at 95% and 99.8% were calculated under a binomial assumption to identify potential outliers while accounting for differences in sample size. Farms falling outside these control limits were considered as exhibiting statistically unusual mortality patterns.

Statistical significance for fixed effects in regression models was set at *P* < 0.05, while farm-level deviations were interpreted based on BLUP CIs and funnel plot limits.

### Evaluation of official control targeting

Official veterinary inspections performed on consignments at the slaughterhouse were summarized descriptively to assess their distribution over the study period, including temporal trends and seasonal variation. For each season, the total number of consignments, the number of inspected consignments, and the corresponding inspection proportion were calculated.

To evaluate whether inspections were preferentially directed towards higher-risk holdings, consignments originating from farms identified as high-risk based on funnel plot analysis were extracted and summarized. The proportion of inspections targeting these farms was calculated.

For inspected consignments, average ambient temperature at loading and estimated transport distance were computed to assess whether inspections were conducted under conditions associated with increased thermal or transport-related stress.

### Software and packages

All statistical analyses were performed using R software (version 4.3.0). The following packages were used as appropriate: lme4, glmmTMB, DHARMa, performance, future.apply, datawizard, dplyr, readxl, and see for mixed-effects modelling and data management; ggplot2, psych, and FSA for descriptive analyses and data visualisation; and logistf, broom, broom.mixed, sandwich, and lmtest for regression modelling and diagnostic procedures.

## Results

### Descriptive analysis

The mean consignment size was 125.4 (SD = 18.0; range: 5–164). Estimated transport distances ranged from 5 to 605 km with an average of 174.2 (SD = 80.9) (Figure S1). Ambient temperatures at loading varied between 0.9 °C and 31.2 °C. All continuous variables showed significant deviations from normality (*P* < 0.001). The number of DOA pigs per consignment ranged from 0 to 7 (mean = 0.077).

The overall mortality proportion was 0.062% (*n* = 2,642 DOA pigs), with values ranging from 0 to 6.897%. Most consignments (93.1%) recorded no deaths (Table S2), resulting in a right-skewed distribution of mortality. Estimated transport distance showed substantial variability, with most journeys concentrated between 100 and 250 km, whereas ambient temperature displayed a wider and bimodal distribution.

Pairwise Spearman’s rank correlations between mortality proportion and continuous predictors (estimated distance, consignment size, and loading temperature) were very weak. Although some associations reached statistical significance due to the large sample size, correlation coefficients were close to zero (*P* ≈ 0–0.07), indicating no meaningful relationships.

Mortality varied significantly across seasons (*P* < 0.001), with the highest values observed in summer (0.0895%), followed by autumn (0.0626%), and the lowest in winter (0.0459%) and spring (0.0473%). Post hoc Dunn’s tests confirmed that summer mortality was significantly higher than in all other seasons. Autumn mortality was significantly lower than in summer (Z = − 6.15) but remained significantly higher than in winter (Z = 4.21) and spring (Z = 3.54). No significant difference was observed between winter and spring (*P* = 1.00) (Table S3).

### Univariable analysis

Univariable logistic regression analyses identified significant associations between several explanatory variables and the odds of mortality at consignment level (Table S4). Ambient temperature at loading was a positive association with mortality, with each 1 °C increase corresponding to an approximate 4% increase in the odds of at least one DOA pig. Consignment size also showed a statistically significant association, with larger consignments associated with higher odds of mortality. In contrast, estimated transport distance was not significantly associated with mortality in the univariable model. Seasonal effects were marked: using autumn as the reference category, the odds of mortality were highest in summer (approximately 35% higher than autumn), whereas winter and spring were associated with significantly lower odds (approximately 26% and 22% lower, respectively, compared to autumn).

### Multivariate analysis and multicollinearity assessment

In the preliminary multivariate Poisson regression of standardized predictors, all covariates were significantly associated with consignment-level mortality (Table S5). Ambient temperature at loading showed the strongest effect (OR = 1.37, 95% CI: 1.31–1.42; *P* < 0.001), indicating a positive association between mortality and higher temperatures. Estimated transport distance was also positively associated with mortality (OR = 1.07, 95% CI: 1.03–1.11; *P* < 0.001), and with consignment size (OR = 1.05, 95% CI: 1.01–1.10; *P* = 0.013).

Correlation analysis among estimated transport distance, consignment size, ambient temperature, and number of DOA pigs showed correlation coefficients that were not significant in all cases (*P* < 0.07) (Figure S2). Variance inflation factors were low for all predictors (VIF < 1.02), indicating no evidence of problematic multicollinearity and supporting the inclusion of these variables in the subsequent mixed-effects models.

### Mixed-effects modelling analysis

All predictors were significantly associated with consignment-level mortality in the mixed-effects model (Table 1). Estimated transport distance (OR = 1.086, 95% CI: 1.012–1.165; *P* = 0.024) and ambient temperature at loading (OR = 1.354, 95% CI: 1.301–1.408; *P* < 0.001) were positively associated with mortality, whereas consignment size showed a negative association (OR = 0.910, 95% CI: 0.865–0.957; *P* < 0.001). Back-transformation of the temperature coefficient indicated that each 1 °C increase in ambient temperature corresponded to a 3.86% increase in the odds of a pig being DOA.

**Table 1:** Mixed-effects logistic regression (GLMM) and zero-inflated binomial (ZIB) model for associaion between covariates and pig mortality during transport 2019–2024

Covariate	Estimate β (GLMM)	Standard error	z value	*P*-value	Odds ratio (GLMM)	Odds ratio (ZIB)
(Intercept)	-7.552	0.042	-181.78	< 0.001	0.0005	0.00126
Distance (z)	0.082	0.036	2.28	0.023	1.086	1.086
Temperature (z)	0.303	0.020	15.02	< 0.001	1.354	1.350
Consignment size (z)	-0.094	0.026	-3.67	0.00024	0.910	0.909

Note: CI = confidence interval; glmer = generalized linear mixed-effects logistic regression; ZIB = zero-inflated binomial model; z = standardized predictor (mean = 0, standard deviation = 1). The estimated farm-level random effect variance is 0.266 (SD = 0.515). The odds ratio for temperature corresponds to a 3.86% increase per 1°C

The random effect of farm accounted for substantial between-farm variability (variance = 0.266; SD = 0.515). Model performance indicated a marginal R² of 0.260 and a conditional R² of 0.934, suggesting that a large proportion of variability was explained by farm-level effects. Residual diagnostics indicated slight overdispersion (dispersion ratio = 1.178; *P* = 0.01) and a limited number of outliers, while overall residual uniformity was acceptable (*P* = 0.022) (Table 2).

**Table 2:** Comparison of fit and diagnostic metrics for GLMM and ZIB models of pig mortality during transport

Metric/Model	GLMM	ZIB
AIC	18,720.1	18,533.9
Degrees of freedom	5	6
Observations	34,239	34,239
Groups (farm_id)	434	434
Conditional R²	0.934	0.930
Marginal R²	0.260	0.270
Dispersion	1.178, *P* = 0.01	1.028, *P* = 0.62
Uniformity test (KS)	D = 0.0081, *P* = 0.022	–
Zero-inflation test	ratioObsSim = 1.006, *P* = 0.114	ratioObsSim = 1.000, *P* = 0.908
Outliers (bootstrap)	34, *P* = 0.04	–

Note: GLMM = generalized linear mixed-effects logistic regression; ZIB = zero-inflated binomial model; AIC: Akaike Information Criterion; R²: coefficient of determination; KS: Kolmogorov–Smirnov test

The ZIB model produced effect estimates consistent with the GLMM and showed improved model fit (AIC = 18,534 vs. 18,720, respectively), with a significant zero-inflation component, indicating an excess of zero-mortality consignments beyond that expected under a standard binomial distribution.

Overall, estimated transport distance and ambient temperature were positively associated with mortality, whereas consignment size showed a modest protective effect.

### Bootstrap-estimated power of model predictors

ORs for all fixed effects in the GLMM and ZIB models are reported in Table 3. Consistent with previous analyses, estimated transport distance and ambient temperature at loading were positively associated with consignment-level mortality, whereas consignment size showed a negative association. Bootstrap-based power analysis indicated high statistical power for ambient temperature (power = 1.00 in both GLMM and ZIB models) and consignment size (0.90 for GLMM; 0.81 for ZIB), supporting the reliability of these effects. Power was 0.42 for GLMM and 0.47 for ZIB for estimated transport distance, reflecting the modest magnitude of its effect. Intercept estimates were highly significant and showed maximal power. Overall, these results support the robustness of the associations identified for ambient temperature and consignment size, while suggesting more cautious interpretation of the effect of estimated transport distance due to its statistical power.

**Table 3:** Odds ratios and bootstrap-estimated statistical power for fixed effects in the GLMM and ZIB models

Predictor	Odds ratio (GLMM)	95% CI	Odds ratio (ZIB)	95% CI	Power (GLMM)	Power (ZIB)
(Intercept)	0.00053	0.00048–0.00057	0.00126	0.00110–0.00144	1.000	1.000
Distance (z)	1.086	1.012–1.165	1.086	1.010–1.166	0.415	0.466
Temperature (z)	1.354	1.301–1.408	1.350	1.293–1.407	1.000	1.000
Consignment size (z)	0.910	0.865–0.957	0.909	0.861–0.958	0.900	0.812

Note: GLMM = generalized linear mixed-effects logistic regression; ZIB = zero-inflated binomial model; CI = confidence interval; z = standardized predictor (mean = 0, standard deviation = 1); statistical power was estimated using parallel bootstrap simulation with 200 resamples

### Farm-level risk categories

**Fig. 2:**
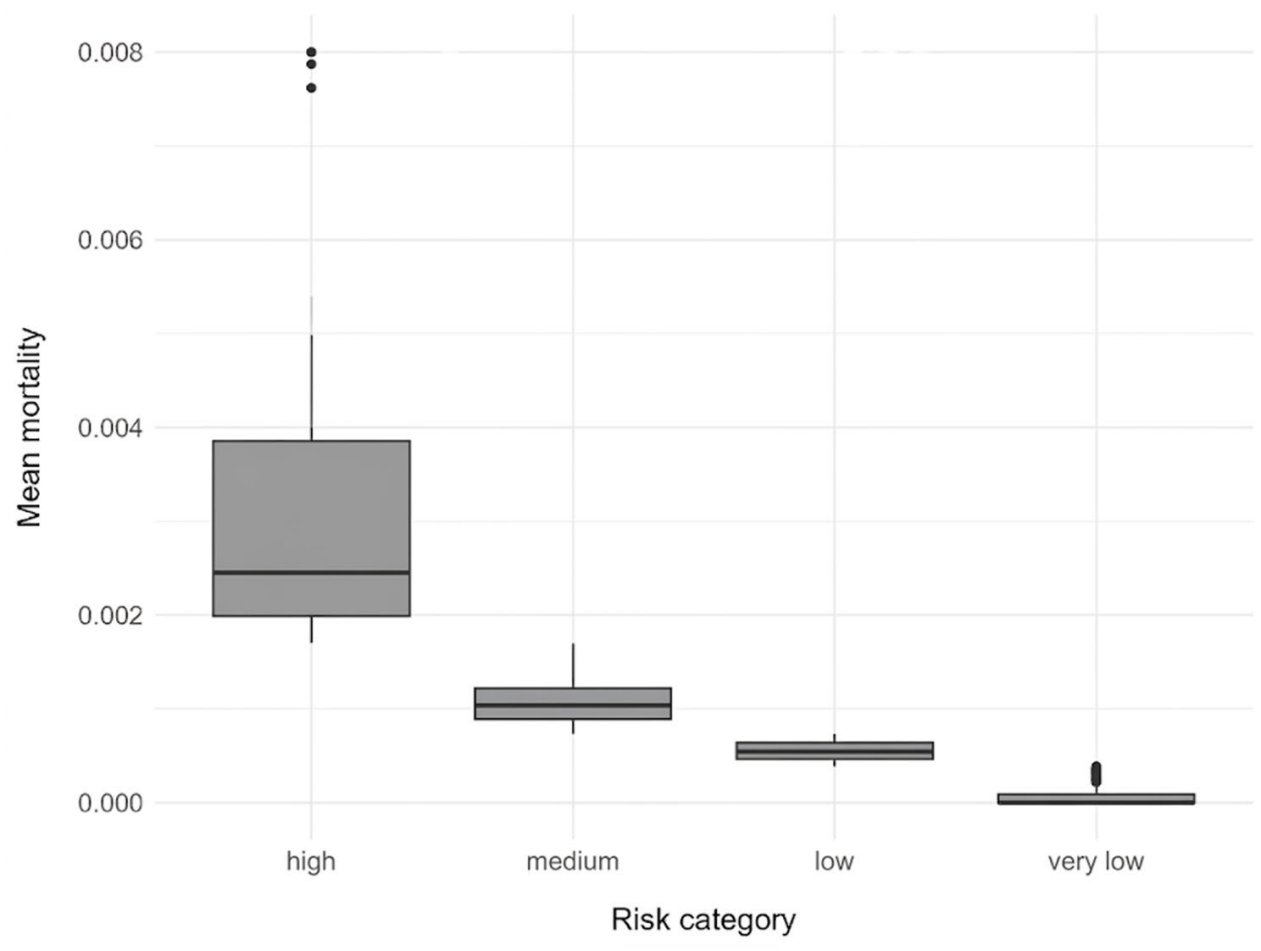
Distribution of average transport mortality per farm across risk categories. Farms were categorized according to mortality distribution percentiles: very low (≤ 25th), low (> 25th to 50th), medium (> 50th to 75th), and high (> 75th percentile). Mortality was calculated as the ratio of total pigs dead-on-arrival (DOA) to the total pigs transported per farm

Average transport mortality per farm ranged from 0% to 0.8%, with a median of 0.0387%, a 25th percentile of 0%, and a 75th percentile of 0.0731%. Farms were classified into four exploratory risk categories (very low, low, medium, and high) based on the 25th, 50th, and 75th percentiles of the mortality distribution. According to these thresholds, 36.9% (*n* = 160) of farms were classified as very low risk, 13.1% (*n* = 57) into low risk, 24.9% (*n* = 108) as medium risk, and 25.1% (*n* = 109) as high risk (Fig. 2).

**Fig. 3:**
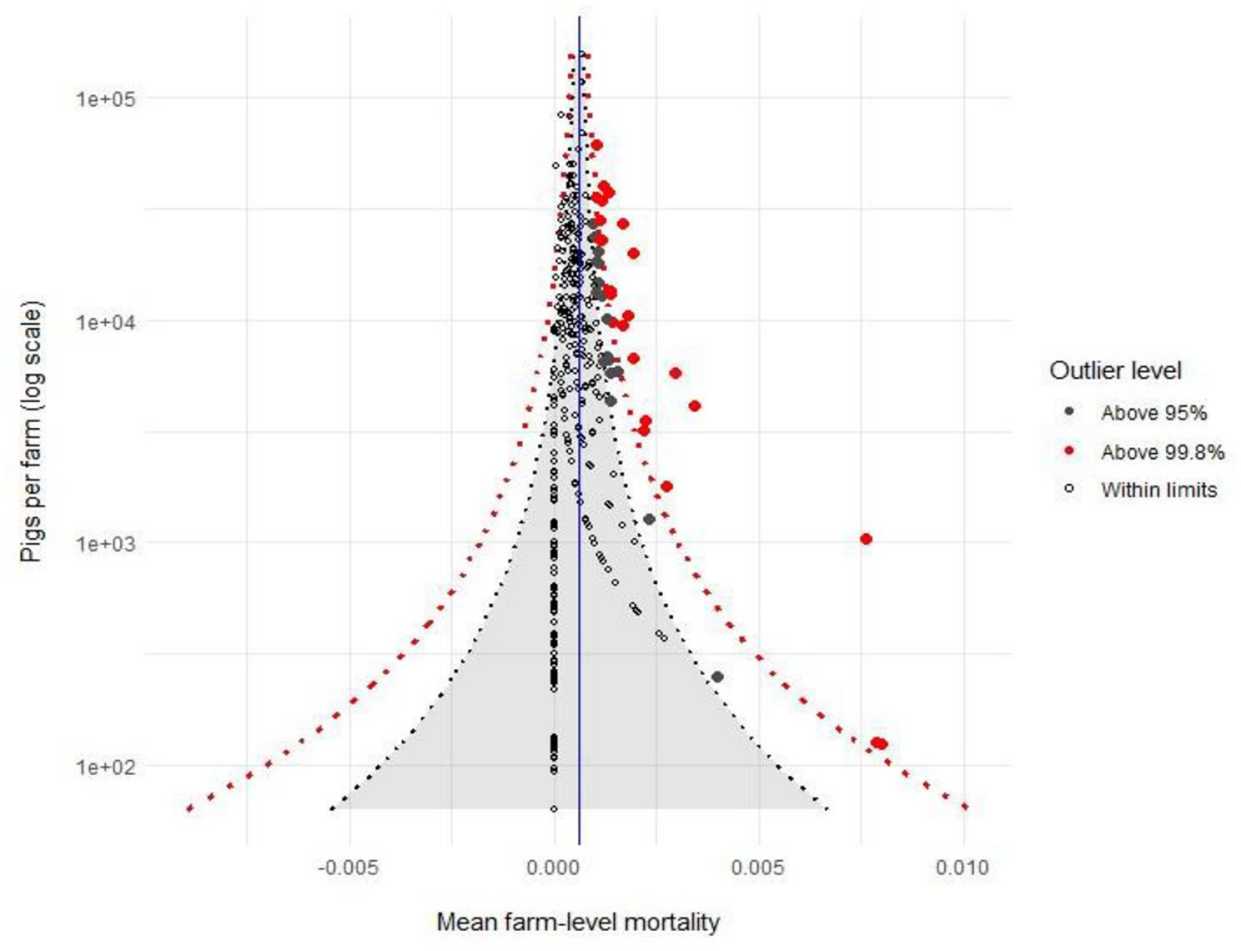
Funnel plot of farm-level mortality, calculated as the percentage of dead-on-arrival (DOA) pigs on the total number of pigs shipped from each farm

BLUPs derived from the GLMM quantified farm-specific deviations from the overall mean mortality risk. Farms with BLUP estimates and corresponding 95% CIs entirely above zero were interpreted as having significantly higher-than-average mortality, identifying 38 farms at elevated risk. Funnel plot analysis, based on 95% and 99.8% binomial control limits, identified 26 farms exceeding the upper 99.8% limit, indicating statistically unusual mortality rates after accounting for the number of pigs transported per farm (Table 4; Fig. 3).

**Table 4:** Farms identified as high-risk outliers in the 99.8% funnel plot of transport mortality

Farm ID	Consignments size	Pigs	Pigs dead (%)	Inspected (%)
28	312	40,085	49 (0.122)	6 (1.92)
33	79	9,758	14 (0.143)	**0** (0.00)
68	26	3,178	7 (0.220)	**0** (0.00)
75	217	27,095	46 (0.170)	6 (2.76)
77	34	4,096	14 (0.342)	**0** (0.00)
80	218	27,904	32 (0.115)	8 (3.67)
86	15	1,810	5 (0.276)	**0** (0.00)
144	1	125	1 (0.800)	**0** (0.00)
168	9	1,050	8 (0.762)	**0** (0.00)
173	279	34,585	41 (0.119)	5 (1.79)
195	162	19,953	39 (0.195)	2 (1.23)
209	55	5,743	17 (0.296)	3 (5.45)
216	490	61,185	63 (0.103)	18 (3.67)
226	282	35,663	37 (0.104)	7 (2.48)
227	107	13,176	17 (0.129)	2 (1.87)
240	1	125	1 (0.800)	**0** (0.00)
247	291	37,214	50 (0.134)	6 (6.87)
260	181	22,813	26 (0.114)	3 (1.66)
261	180	22,700	27 (0.119)	8 (4.44)
277	33	3,553	8 (0.225)	3 (9.09)
318	85	10,552	19 (0.180)	1 (1.18)
344	1	127	1 (0.787)	**0** (0.00)
361	77	9,406	16 (0.170)	1 (1.30)
374	55	6,730	13 (0.193)	2 (3.64)
404	106	13,044	18 (0.138)	2 (1.89)
420	109	13,580	19 (0.140)	3 (2.75)

### Veterinary inspections and risk-based targeting

A total of 664 out of 34,239 consignments (1.95%) underwent official veterinary inspection. For the inspected consignments, the mean consignment size was 127.2 pigs (SD = 16.9, range: 18–155), the average estimated transport distance was 171.3 km (SD = 78.6, range: 15–390), and ambient temperatures at loading ranged from 1.8 °C to 29.7 °C. The number of pigs DOA per consignment ranged from 0 to 2 (mean = 0.045), corresponding to a mortality proportion of 0–2% (mean = 0.036%). Most inspected consignments (95.6%) recorded no deaths (Table S6).

**Fig. 4:**
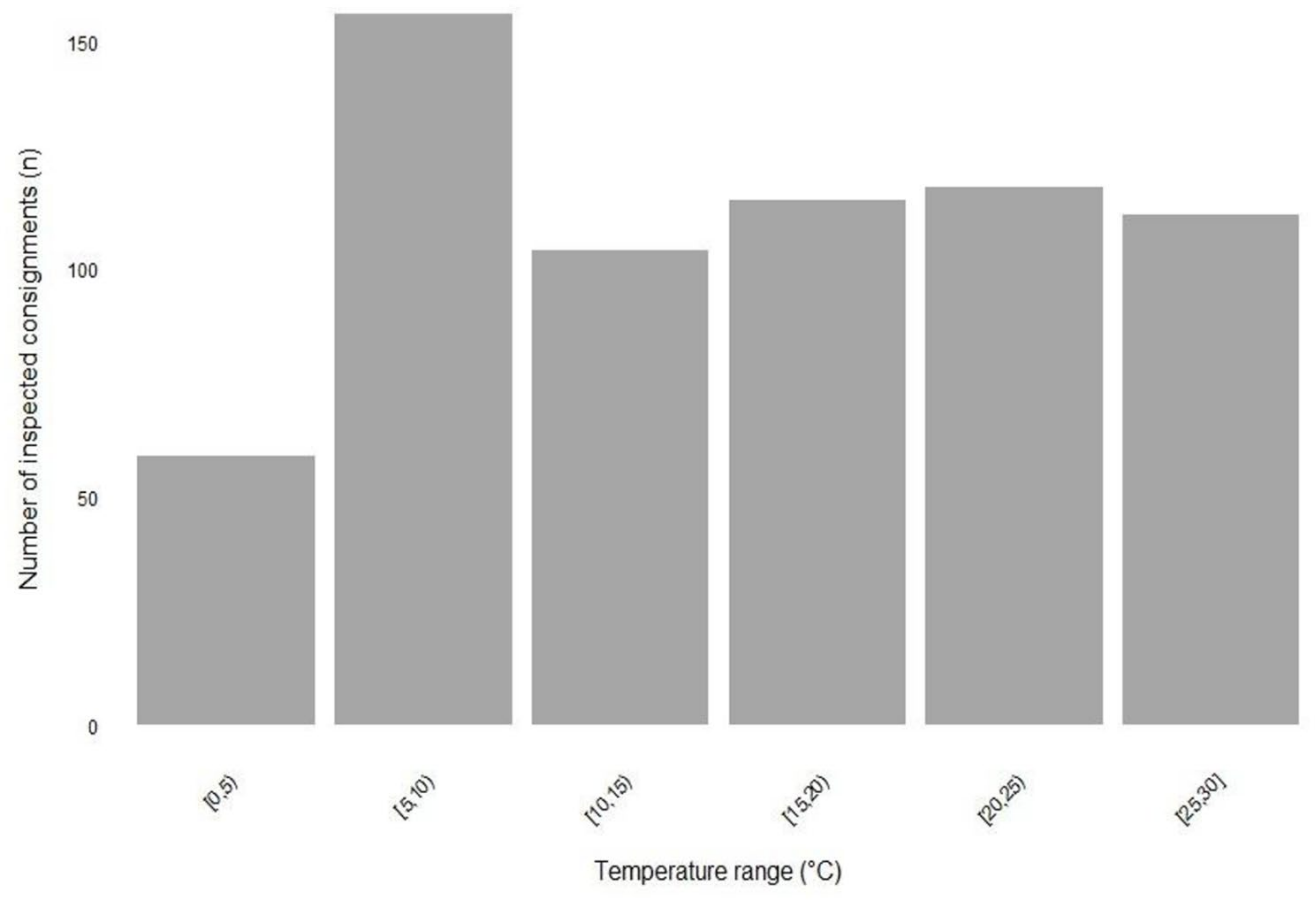
Number of veterinary inspections of pig consignments (*n* = 664) by ambient temperature at loading

Inspection coverage appeared consistent across temporal and environmental conditions. When stratified by season, inspections were relatively evenly distributed: 22.0% (*n* = 146) in winter, 26.0% (*n* = 173) in spring, 25.5% (*n* = 169) in summer, and 26.5% (*n* = 176) in autumn. Similarly, inspections were distributed across the full range of ambient temperatures (0–30 °C) (Fig. 4), with no evident prioritisation of consignments based on consignment size, estimated transport distance, or observed mortality.

To evaluate the targeting of high-risk farms, consignments originating from farms identified as outliers above the 99.8% control limit in the funnel plot were examined. Among the 26 high-risk farms, the number of consignments per farm varied widely (range: 1–490), and the proportion of inspected consignments ranged from 0% to 9.1% (Table 4). Overall, only 12.95% (*n* = 86) of inspected consignments originated from these high-risk farms. While some high-risk farms were inspected multiple times, others (30.8%) with consistently elevated mortality were not inspected during the study period.

## Discussion

This retrospective observational study provides a comprehensive assessment of factors associated with transport-related mortality in pigs delivered to a high-throughput Italian slaughterhouse, and highlights important limitations in current regulatory enforcement. The findings support the potential value of a risk-based enforcement (RBE) approach to improve both animal welfare and the efficiency of official controls.

The overall mortality rate (0.062%) was consistent with previous EU reports [6]. However, mortality represents only the most extreme outcome of transport-related stress and does not necessarily reflect the full extent of welfare compromise, as sub-lethal stressors remain undetected. Although a slight upward trend was observed over time, values remained within the range reported in the literature [32, 33]. Despite relatively low mortality rates, transport remains a major contributor to non-compliance with animal welfare legislation and a relevant source of economic loss [34].

Ambient temperature emerged as the main factor associated with mortality, confirming that current practices are insufficient to protect pigs under warm climatic conditions. Mortality increased during summer and decreased in winter and spring, consistent with the well-documented susceptibility of pigs to heat stress [22, 24, 32, 33, 35]. The estimated 3.86% increase in mortality odds for each 1 °C rise at loading highlights a progressive and biologically meaningful risk gradient. These findings reinforce the need for mitigation strategies such as adjusted loading schedules, improved ventilation systems, and heat-adapted transport protocols, particularly during warmer periods. Studies on heavy pigs in PDO systems similarly highlight their increased sensitivity to heat and handling compared to lighter, conventional pigs [22, 25, 35, 36].

Estimated transport distance was also positively associated with mortality, indicating that transport-related stress accumulates even within journeys lasting ≤ 8 h. This finding does not support the hypothesis of a protective or recovery phase during transport. Rather, it aligns with recent experimental evidence showing progressive deterioration in clinical condition and increased fatigue as journey duration increases [18, 19]. Although the magnitude of this effect was modest and associated with lower statistical power, it remains biologically plausible and consistent with cumulative stress exposure during transport.

Interestingly, larger consignment sizes were associated with a slightly lower mortality risk. This finding may reflect indirect effects related to vehicle characteristics and management practices, as larger consignments are more likely to be transported using modern vehicles equipped with improved ventilation and suspension systems. Furthermore, the benefit of larger consignments may stem from a tighter logistical synchronization between farm housing units and vehicle compartments. When the number of pigs in a farm pen matches the capacity of a truck compartment, the need for regrouping or mixing unfamiliar individuals is minimized. This reduces the incidence of agonistic interactions and social instability, which are known stressors regardless of vehicle technology. Thus, the association between larger consignments and lower mortality may be mediated by the maintenance of social cohesion enabled by compatible farm-to-truck infrastructure, rather than group size per se. While stocking density was not directly assessed, it remains a well-established determinant of welfare outcomes [5, 37, 38]. The observed association suggests that batch size may act as a proxy for transport quality, warranting further investigation.

Substantial between-farm variability was observed, highlighting the importance of farm-level factors in determining transport outcomes. The classification of farms into risk categories and the identification of high-risk holdings through BLUP estimates and funnel plot analysis provide a robust and complementary framework for benchmarking performance and prioritising interventions. Transport mortality can be interpreted as a “biological indicator” of pre-transport fitness, reflecting underlying differences in health status, management, and biosecurity. Although transport outcomes result from the interaction between farm conditions and journey-related factors, the consistent identification of a subset of high-risk farms underscores the relevance of farm-level monitoring within a risk-based control system. However, as farms may consistently use the same transport operators, further research is needed to disentangle farm and transport effects within the supply chain.

The Italian ClassyFarm system already provides a structured framework for farm-level risk assessment based on animal health, welfare, and antimicrobial use [39], but currently does not incorporate transport-related outcomes such as DOA. The integration of slaughterhouse-derived indicators into this system would strengthen its capacity to identify high-risk farms and support data-driven enforcement strategies. In this context, the slaughterhouse represents a critical control point where animal-based outcomes can be systematically recorded and linked back to farms and transport conditions.

Despite this potential, the present study shows that official controls are not currently aligned with risk. Fewer than 2% of consignments were inspected, and inspections were evenly distributed across seasons, temperatures, and consignment characteristics. Importantly, only 12.95% of inspected consignments originated from high-risk farms, and a substantial proportion of these farms were never inspected. Furthermore, inspected consignments showed lower mortality compared to non-inspected ones, indicating that inspections are not preferentially targeting higher-risk situations. These findings suggest inefficient allocation of veterinary resources and a missed opportunity to improve welfare outcomes through targeted monitoring. In addition, integrating predictive metrics at the farm and batch levels could enhance both the effectiveness and efficiency of inspections, provided that transport mortality is associated with on-farm factors.

The current regulatory framework, based on Council Regulation (EC) No 1/2005 [8] and its implementation through the Italian PNBA [9], relies largely on prescriptive measures and minimum inspection quotas. This approach may be inadequate under conditions of increasing climatic variability, particularly in Southern Europe. For journeys lasting ≤ 8 h, the absence of specific requirements for microclimate control (e.g. active cooling systems) limits the effectiveness of welfare protection during heat stress events. Moreover, fixed inspection percentages (e.g. 2% or 10%) may result in the inspection of low-risk consignments while high-risk ones remain unchecked, conflicting with the principles of efficient risk-based regulation.

The findings of this study support a transition from compliance-based controls towards a proactive RBE model. Such an approach could in theory prioritise inspections based on key risk indicators, including ambient temperature and farm-level risk profiles, potentially supported by real-time weather data and historical performance metrics. Within this framework, high-risk consignments could be systematically targeted, while low-risk consignments would be subject to reduced or random inspection. This strategy would align with the existing ClassyFarm infrastructure and improve both the effectiveness and efficiency of official controls.

Several limitations should be acknowledged. Estimated transport distance was used as a proxy for journey duration, which may introduce imprecision in exposure assessment. The lack of direct measurements of in-vehicle microclimatic conditions is another limitation, as ambient temperature may not fully reflect the thermal environment experienced by pigs. In addition, other relevant factors such as handling practices, vehicle characteristics, and group composition were not available. Future studies incorporating real-time environmental monitoring and detailed transport data would allow for more accurate risk modelling. Nonetheless, the modelling approach adopted in this study appropriately accounted for the hierarchical structure of the data, with farm included as a random effect, and addressed the high prevalence of zero-mortality consignments through the use of a zero-inflated model.

## Conclusions

The results of this study indicate that mortality during pigs’ journeys lasting ≤ 8 h is primarily associated with ambient temperature and season, linked to journey duration, and lower when larger consignment sizes are used. Although most farms exhibit minimal mortality during transport, a subset consistently showed elevated risk. To enhance animal welfare during transport, veterinary inspections could be aligned with these risk patterns, highlighting the potential of farm-level, data-driven targeting of inspections to optimize welfare monitoring and regulatory strategies. These findings provide practical guidance for planning inspections, scheduling transport, and implementing risk-based control measures aimed at reducing pre-slaughter mortality in heavy pigs.

## Supplementary Information

Below is the link to the electronic supplementary material.

Supplementary Material 1

Supplementary Material 2

## Data Availability

The datasets used and/or analyzed during the current study are available from the corresponding author upon reasonable request.
