# Supplementary material for: Patterns and risk factors of pig transport mortality: implications for official controls in a high-throughput slaughterhouse
Source: Porcine Health Manag. 2026 May 7;12:39. doi: 10.1186/s40813-026-00519-z (PMC13321726; doi:10.1186/s40813-026-00519-z)

**Figure S1.** Distribution of consignments by transport distance and ambient temperature.

**
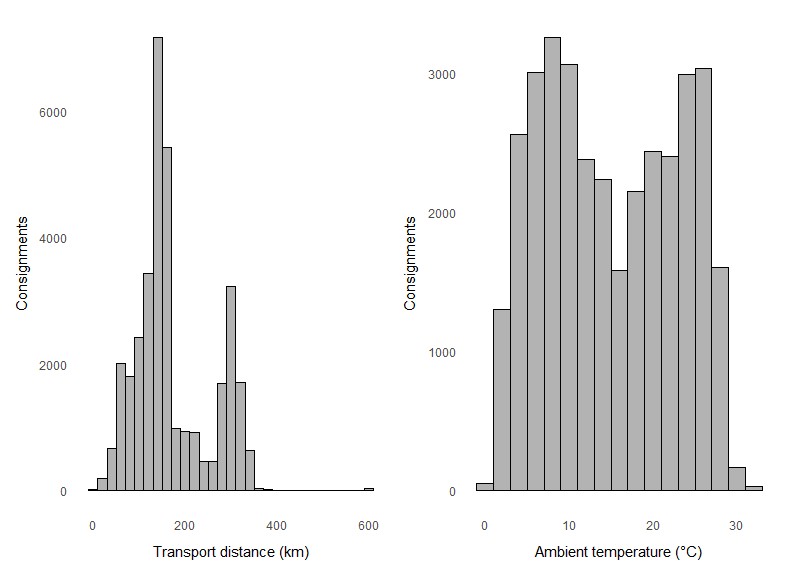
**

**Figure S2.** Correlation matrix (Spearman correlation coefficient) of between mortality proportion and continuous predictors.


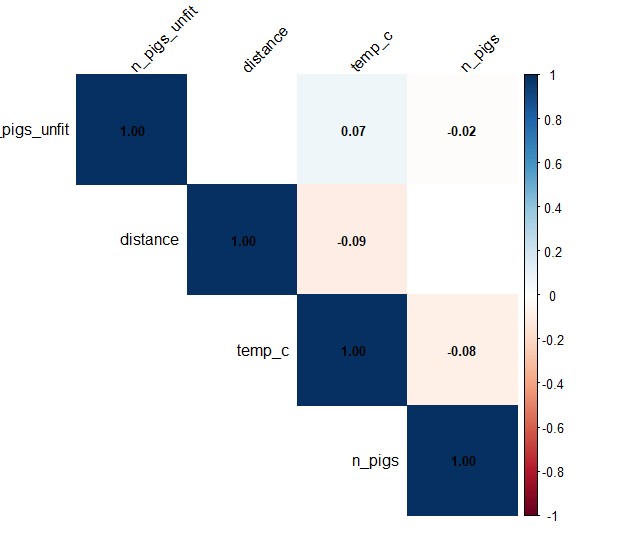

Supplement: Supplementary file 1 — Supplementary Material 1 [file 40813_2026_519_MOESM1_ESM.docx]
