# Supplementary material for: Patterns and risk factors of pig transport mortality: implications for official controls in a high-throughput slaughterhouse
Source: Porcine Health Manag. 2026 May 7;12:39. doi: 10.1186/s40813-026-00519-z (PMC13321726; doi:10.1186/s40813-026-00519-z)
Supplement: Supplementary file 2 — Supplementary Material 2 [file 40813_2026_519_MOESM2_ESM.docx]

**Table S1.** List of variables included in the study.

| Variable | Description | Type |
| --- | --- | --- |
| Farm | Farm of origin of each consignment. Included as a random effect to account for clustering of consignments within farms. | Categorical |
| Season | Season in which each consignment was transported, categorized into the four meteorological seasons (spring, summer, autumn, winter). | Categorical |
| Mortality | Proportion of animals that died during transport for each consignment. | Continuous |
| Distance | The distance from each farm to the slaughterhouse was calculated selecting the shortest available road route | Continuous |
| Temperature | Ambient temperature at loading the pigs, defined as the average monthly temperature at the farm location, obtained from official meteorological records. | Continuous |
| Consignment size | Number of pigs per consignment, obtained from slaughterhouse and derived from routine veterinary activities. | Continuous |

**Table S2.** Descriptive analysis of consignments by season, farm, and zero-mortality.

| Variable | Category | No | % |
| --- | --- | --- | --- |
| Season | Autumn | 8,129 | 23.7 |
|  | Summer | 8,896 | 26.0 |
|  | Winter | 8,398 | 24.5 |
|  | Spring | 8,816 | 25.7 |
| Farm | Farms | 434 | – |
|  | Median consignments per farm | 40 | – |
|  | Min–Max consignments per farm | 1–1,215 | – |
| Mortality | Zero deaths | 31,891 | 93.1 |

**Table S3.** Pairwise comparisons of pig transport mortality across seasons with Dunn’s post-hoc test and Bonferroni correction.

| Comparison | Z-score | P.adj | Significant |
| --- | --- | --- | --- |
| Autumn – Summer | -6.15 | 4.57 × 10⁻⁹ | Yes |
| Autumn – Winter | 4.21 | 1.53 × 10⁻⁴ | Yes |
| Summer – Winter | 10.51 | 4.67 × 10⁻²⁵ | Yes |
| Autumn – Spring | 3.54 | 2.43 × 10⁻³ | Yes |
| Summer – Spring | 9.90 | 2.48 × 10⁻²² | Yes |
| Winter – Spring | -0.73 | 1.00 | No |

**Note:** P.adj = adjusted P-value; Yes = P < 0.05, No = P ≥ 0.05.

**Table S4.** Univariable logistic regression of predictors associated with mortality in pig consignments (n = 34,239).

| Predictor | Odds Ratio | 95% CI | P-value |
| --- | --- | --- | --- |
| Distance (km) | 1 | 1-1 | 0.14 |
| Temperature (°C) | 1.04 | 1.03-1.04 | 0 |
| Consignment size (No) | 1 | 1-1.01 | 0.001 |
| Summer | 1.35 | 1.21-1.5 | 0 |
| Winter | 0.74 | 0.65-0.84 | 0 |
| Spring | 0.78 | 0.69-0.89 | 0 |

**Note:** CI = confidence interval.

**Table S5.** Preliminary multivariate Poisson regression of pig mortality during transport (without farm-level random effects).

| Covariate | Estimate β | Standard error | z value | P-value | Odds ratio | 95% CI |
| --- | --- | --- | --- | --- | --- | --- |
| (Intercept) | 0.0734 | 0.0204 | -128.0 | <0.001 | 0.073 | 0.071–0.076 |
| Distance (z) | 0.067 | 0.0193 | 3.67 | 0.000247 | 1.07 | 1.03–1.11 |
| Temperature (z) | 0.314 | 0.0200 | 15.6 | <0.001 | 1.37 | 1.31–1.42 |
| Consignment size (z) | 0.049 | 0.0211 | 2.49 | 0.0129 | 1.05 | 1.01–1.10 |

**Note:** CI = confidence interval; z = standardized predictor (mean = 0, standard deviation = 1).

**Table S6**. Descriptive analysis of consignments inspected (n = 664) by season, farm, and zero-mortality.

| Variable | Category | Consignments inspected | % |
| --- | --- | --- | --- |
| Season | Autumn | 176 | 26.5 |
|  | Summer | 169 | 25.5 |
|  | Winter | 146 | 22.0 |
|  | Spring | 173 | 26.1 |
| Farm | Farms | 200 | – |
|  | Median consignments per farm | 2 | – |
|  | Min–Max consignments per farm | 1–26 | – |
| Mortality | Zero deaths | 635 | 95.6 |

**Note:** For farm rows, percentages are not calculated since these are descriptive statistics of farms, not consignments.
